# Stevioside Ameliorates Palmitic Acid–Induced Abnormal Glucose Uptake via the PDK4/AMPK/TBC1D1 Pathway in C2C12 Myotubes

**DOI:** 10.1002/edm2.482

**Published:** 2024-03-31

**Authors:** Changfa Zhang, Shuai Li, Likang Li, Ruoting Wang, Shiming Luo, Guowei Li

**Affiliations:** ^1^ Center for Clinical Epidemiology and Methodology (CCEM) Guangdong Second Provincial General Hospital Guangzhou China; ^2^ Fertility Preservation Lab, Guangdong‐Hong Kong Metabolism and Reproduction Joint Laboratory, Reproductive Medicine Center Guangdong Second Provincial General Hospital Guangzhou China; ^3^ Department of Health Research Methods, Evidence, and Impact (HEI) McMaster University Hamilton Ontario Canada

**Keywords:** glucose metabolism, PDK4, stevioside, sweetener

## Abstract

**Background:**

Stevioside (SV) with minimal calories is widely used as a natural sweetener in beverages due to its high sweetness and safety. However, the effects of SV on glucose uptake and the pyruvate dehydrogenase kinase isoenzyme (PDK4) as an important protein in the regulation of glucose metabolism, remain largely unexplored. In this study, we used C2C12 skeletal muscle cells that was induced by palmitic acid (PA) to assess the effects and mechanisms of SV on glucose uptake and PDK4.

**Methods:**

The glucose uptake of C2C12 cells was determined by 2‐NBDG; expression of the *Pdk4* gene was measured by quantitative real‐time PCR; and expression of the proteins PDK4, p‐AMPK, TBC1D1 and GLUT4 was assessed by Western blotting.

**Results:**

In PA‐induced C2C12 myotubes, SV could significantly promote cellular glucose uptake by decreasing PDK4 levels and increasing p‐AMPK and TBC1D1 levels. SV could promote the translocation of GLUT4 from the cytoplasm to the cell membrane in cells. Moreover, in *Pdk4*‐overexpressing C2C12 myotubes, SV decreased the level of PDK4 and increased the levels of p‐AMPK and TBC1D1.

**Conclusion:**

SV was found to ameliorate PA‐induced abnormal glucose uptake via the PDK4/AMPK/TBC1D1 pathway in C2C12 myotubes. Although these results warranted further investigation for validation, they may provide some evidence of SV as a safe natural sweetener for its use in sugar‐free beverages to prevent and control T2DM.

## Graphical abstract

Stevioside could improve palmitic acid–induced abnormal glucose uptake via the PDK4/AMPK/TBC1D1 pathway in C2C12 myotubes, supporting the use of SV as a safe natural sweetener in sugar‐free beverages to prevent and control T2DM.

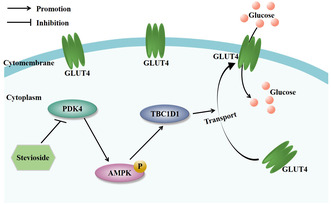

## 1 Introduction

Type 2 diabetes mellitus (T2DM) remains a severe public health issue globally, with an expected amount of approximately 500 million in 2021 [1]. Unhealthy diet is a major risk factor contributing to T2DM [2], especially the long‐term consumption of high‐calorie foods and drinks [3]. To restrict excessive intake of energy, sugar‐free beverages with sweetness have been emergingly used worldwide [4]. Sugar‐free beverages are generally made with no‐ or low‐calorie sweeteners instead of sucrose [5, 6], where the sweeteners can be categorised as either artificial or natural [7].

There is a growing body of evidence suggesting that artificial sweeteners may increase the risks of cancers, cardiovascular diseases and glucose intolerance [8, 9, 10, 11]. In contrast, stevioside (SV), as a natural sweetener with no safety concerns reported so far [12], is 250–300 times sweeter than sucrose but has minimal calories [13]. Previous preclinical studies have found that SV may improve insulin resistance and glucolipid metabolism [14, 15, 16]. Nevertheless, few other studies reported no significant effect of SV on glucose uptake [17]. Therefore, more explorations are needed to further assess the impact and molecular mechanism of SV on glucose uptake.

Pyruvate dehydrogenase kinase isozyme 4 (PDK4) has been recognised as an important protein in the regulation of glucose metabolism [18]. It has been shown that the expression of PDK4 is significantly upregulated in skeletal muscles of patients with insulin resistance [19]. PDK4 can inhibit the activity of the pyruvate dehydrogenase (PDH) complex through phosphorylation, thereby regulating glucose uptake in muscles [20]. As the most important organ for maintaining systemic glucose homeostasis, abnormal functions of skeletal muscles could substantially impair glycaemic control and increase the risk of T2DM [21, 22]. However, the relationship between SV, PDK4 and glucose uptake in skeletal muscles remains unknown.

In this in vitro study, we used C2C12 cells of skeletal muscles from mice to investigate the effects of SV on glucose uptake and PDK4. Results from our study may generate some evidence of SV regarding its use in sugar‐free beverages for the prevention and control of T2DM.

## 2 Materials and Methods

### 2.1 Cell Culture

C2C12 cells (STCC20027G; Servicebio, China) were cultured in Dulbecco's modified eagle medium (DMEM, C11995500BT; Gibco) supplemented with 10% (v/v) foetal bovine serum (FBS, AUS‐01S‐02; Cell‐Box) and 1% (v/v) penicillin–streptomycin solution (15140‐122; Gibco) in an incubator (37°C and 5% CO_2_). Upon reaching 80% density, cells were differentiated for 3 d with DMEM culture solution containing 2% horse serum. Subsequently, the cells were manipulated according to various experimental requirements.

### 2.2 Reagent Preparation and Plasmid Construction

According to the literature, we chose the concentration of 0.4 mM (mmol/L) for palmitic acid (PA) [23, 24, 25]. To prepare PA, we first dissolved sodium palmitate (P9767; Sigma‐Aldrich) in 1× PBS at 70°C, where 10% of fatty acid–free bovine serum albumin (BSA, A8850; Solarbio) was prepared in 1× PBS. Both solutions were mixed and conjugated at 55°C and then diluted with DMEM to obtain the required final concentration. According to the previous reports, the final molecular ratio of free fatty acid to BSA was 5.2:1 [26]. SV (HY‐N0669, MCE) was prepared as a 40 mM concentrated reservoir solution with ultrapure water and set aside. Sodium dichloroacetate (DCA) as an inhibitor of PDK4 was made into a 50 mM concentrated reservoir with ultrapure water and finally diluted to a 0.5 mM working solution with the culture medium for further treatment.

We conducted the *Pdk4*‐overexpressing plasmid as follows. First, the mEmerald‐Mito‐7 (#54160; Addgene) plasmid was digested using *Nhe*I with *Not*I. The CDS sequence of the *Pdk4* gene (NCBI, NM_013743.2) was ligated to the mOrange sequence using T2A (sequence: GGAAGCGGAGAGGGCAGGGGAAGTCTTCTAACATGCGGGGACGTGGAGGAAAATCCCGGCCCC). Sequences were synthesised by Genewiz Biotechnology Ltd. (Suzhou, China), and the target fragments were recombined with linearised vectors using the ClonExpress Ultra One Step Cloning Kit (C115; Vazyme, China).

### 2.3 Glucose Uptake Assay

C2C12 cells were inoculated in 6‐well plates and cultured for differentiation for 3 d. Then, the cultured cells were treated in the following ways: (1) treated with 0.4 mM PA for various times (0, 6, 12 and 24 h); (2) co‐treated with different concentrations of SV (0.25, 0.5, 0.75 and 1 mM) with 0.4 mM PA for 24 h; (3) co‐treated with 0.5 mM DCA with 0.4 mM PA for 24 h; and (4) transfected with the *Pdk4*‐overexpressing plasmid using the Lipo6000 transfection reagent (C0526FT; Beyotime, China) for 24 h. The normal control group was the C2C12 cells treated with BSA alone.

Subsequently, the cells were starved with low‐glucose medium for 2 h and cultured with 1× PBS containing 100 μM 2‐NBDG (GC10289; GlpBio) for 30 min [27]. To measure the insulin‐stimulated glucose uptake, cells were first treated with 1× PBS diluted with 100 nM insulin (P3376‐100IU; Beyotime, China) for 30 min, followed by treatment with 100 μM 2‐NBDG for 30 min. Finally, the cells were washed with DMEM twice and replaced with a new medium. The fluorescence detection and analysis was performed by the flow cytometry (CytoFLEX; Beckman Coulter, USA) to quantify the cells' glucose uptake.

### 2.4 Cell Viability

Cells were differentiated as described above and treated with BSA, 0.4 mM PA and 0.4 mM PA plus 0.75 mM SV for 24 h, respectively. Following this, in each well 100 μL of fresh culture solution was replaced and then incubated for 2 h with 10 μL of CCK‐8 solution (A311; Vazyme, China) per well. Absorbance at 450 nm was detected by an enzyme‐labelled instrument (Multiskan Sky; Thermo Scientific) to measure the cell viability.

### 2.5 Quantitative Real‐Time PCR (qRT‐PCR)

Total RNA was extracted from cell samples using the Total RNA Isolation Kit (RC101; Vazyme, China) according to the manufacturer's instructions. Reverse transcription was performed using 1 μg RNA as described in the cDNA Synthesis Kit (R323; Vazyme, China). The transcript levels of genes between groups were analysed using SYBR qPCR Master Mix (Q711; Vazyme, China) on the ABI7500 Real‐Time System. Data were normalised with *beta‐actin* and analysed by $2^{-\Delta \Delta C_{t}}$. Table 1 shows the sequences of all primers used in this study.

**TABLE 1:** Sequences of all primers for qRT‐PCR.

Primers	Sequences	GenBank ID
*beta‐actin*—Forward	5′‐GGCTGTATTCCCCTCCATCG‐3′	NM_007393.5
*beta‐actin*—Reverse	5′‐CCAGTTGGTAACAATGCCATGT‐3′	
*Pdk4*—Forward	5′‐TCCGAAGCTGATGACTGGTG‐3′	NM_013743.2
*Pdk4*—Reverse	5′‐ACAGACCCACTTTGATCCCG‐3′	
*Prkaa1*—Forward	5′‐GTCAAAGCCGACCCAATGATA‐3′	NM_001013367.3
*Prkaa1*—Reverse	5′‐CGTACACGCAAATAATAGGGGTT‐3′	
*Prkaa2*—Forward	5′‐CAGGCCATAAAGTGGCAGTTA‐3′	NM_001356568.1
*Prkaa2*—Reverse	5′‐AAAAGTCTGTCGGAGTGCTGA‐3′	

### 2.6 Western Blotting Analysis

Cell samples were lysed with RIPA lysis buffer (P0013K; Beyotime, China) containing protease and phosphatase inhibitor cocktail (P1045; Beyotime, China) for 30 min on ice, when the cells were vortexed and mixed for every 5 min. Cell membrane proteins and cytoplasmic proteins were extracted using the Mammalian Membrane Protein Extraction Kit (DE301‐01; TransGen, China). The protein concentration was determined by BCA Protein Assay Kit (P0012S; Beyotime, China). The separation gel of 10%–12% was prepared with the SDS‐PAGE Gel Preparation Kit (P0012AC; Beyotime, China). After adding 20–40 μg of protein sample per well, electrophoresis was performed with Tris–Gly electrophoresis buffer in electrophoresis apparatus (PowerPac Basic; Bio‐Rad, USA). Subsequently, the proteins were transferred to the PVDF membrane (IPVH00010; Merck Millipore) by a membrane transfer apparatus (PowerPac Basic; Bio‐Rad, USA). Blocking was done with 5% skim milk. After the primary and secondary antibodies were incubated, the protein contents were detected by chemiluminescence with BeyoECL Star (P0018AS; Beyotime, China). In order to avoid errors due to re‐sampling and to facilitate the subsequent analysis of the results, we used a special stripping buffer (P0025N; Beyotime, China) to remove the primary and secondary antibodies from the PVDF membrane and to block and re‐incubate new antibodies. The integrated density of all protein images was analysed and measured by the ImageJ software.

Antibodies included the following: actin (1:1000, 20536‐1‐AP; Proteintech Group, China), PDK4 (1:1000, 12949‐1‐AP; Proteintech Group, China), phospho‐AMPK (1:1000, p‐AMPK, AF5908; Beyotime, China), AMPK (1:1000, AF6195; Beyotime, China), TBC1D1 (1:1000, 22124‐1‐AP; Proteintech Group, China), GLUT4 (1:1000, AF6999; Beyotime, China), ATP1A1 (1:1000, 14418‐1‐AP; Proteintech Group, China) and HRP‐conjugated goat anti‐rabbit IgG (1:2000, A0208; Beyotime, China).

### 2.7 Statistical Analyses

Data were described as mean ± standard deviation (SD). We analysed the overall data by one‐way ANOVA and performed pairwise comparisons by Dunnett's test, both with a *p* < 0.05 indicating statistical significance. All statistical analyses were performed using GraphPad Prism 8.

## 3 Results

### 3.1 PA Reduced Glucose Uptake in C2C12 Myotubes While SV Could Enhance Glucose Uptake

**FIGURE 1:**
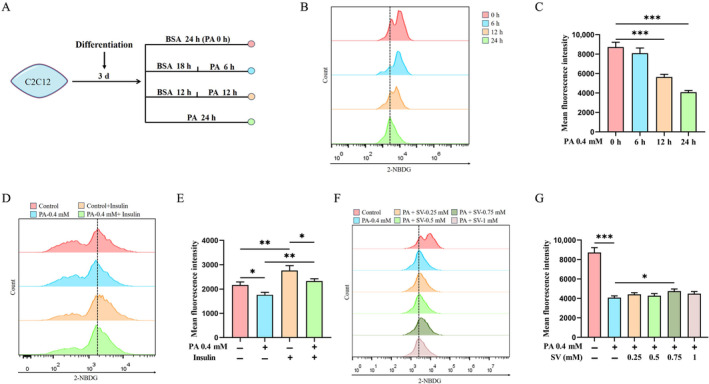
PA reduced glucose uptake in C2C12 myotubes while SV could enhance glucose uptake. (A) Flow diagram showing the treatment of C2C12 myoblasts (PA concentration as of 0.4 mM). (B) Glucose uptake capacity of C2C12 myotubes (differentiated for 3 d) at different time points of 0.4 mM PA treatment was measured by 2‐NBDG, a fluorescent deoxyglucose derivative. (C) Quantitative analysis of the mean fluorescence intensity of 2‐NBDG uptake of C2C12 myotube at different time points of 0.4 mM PA treatment. (D) The glucose uptake capacity of C2C12 myotubes was measured with 2‐NBDG in the presence of insulin stimulation after 24‐h treatment with 0.4 mM PA. (E) Quantitative analysis of the mean fluorescence intensity of 2‐NBDG uptake by insulin‐stimulated C2C12 myotubes (0.4 mM PA treatment for 24 h). (F) Glucose uptake capacity of C2C12 myotubes that were co‐treated with different concentrations of SV and 0.4 mM PA for 24 h was examined by 2‐NBDG. (G) Quantitative analysis of the mean fluorescence intensity of 2‐NBDG uptake of C2C12 myotubes that were co‐treated with different concentrations of SV and PA for 24 h. Each experiment was performed with three replications. Data are expressed as mean ± SD (*n* = 3). *p < 0.05, **p < 0.01, ***p < 0.001.

C2C12 cells that had been differentiated for 3 d were treated with 0.4 mM PA. The ability of the C2C12 myotubes to uptake glucose from the medium gradually diminished with the duration of PA treatment (Figure 1A–C). Because the 24‐h‐treated group had the smallest glucose uptake when compared with the control group, we used the treatment of PA for 24 h to construct the *model* of lipid‐induced abnormal cellular glucose uptake in C2C12 myotubes (i.e., the PA‐alone‐treated group for 24 h as the *model*). Glucose uptake was significantly reduced in insulin‐stimulated C2C12 myotubes treated with PA for 24 h compared with the control group (Figure 1D,E), indicating the successful *model* of insulin resistance of C2C12 myotubes.

Subsequently, C2C12 myotubes differentiated for 3 d were co‐treated with different concentrations of SV (0.25, 0.5, 0.75 and 1 mM) plus 0.4 mM PA. It was found that the 0.75 mM of SV had the greatest effect on promoting glucose uptake when compared with the PA‐alone‐treated group (4078 ± 177.9 vs. 4742 ± 222.9 A.U., *p* = 0.016, Figure 1F,G). Therefore, 0.75 mM SV was used to treat the cells in all subsequent experiments.

### 3.2 SV Reduced PDK4 Expression in PA‐Induced C2C12 Myotubes

**FIGURE 2:**
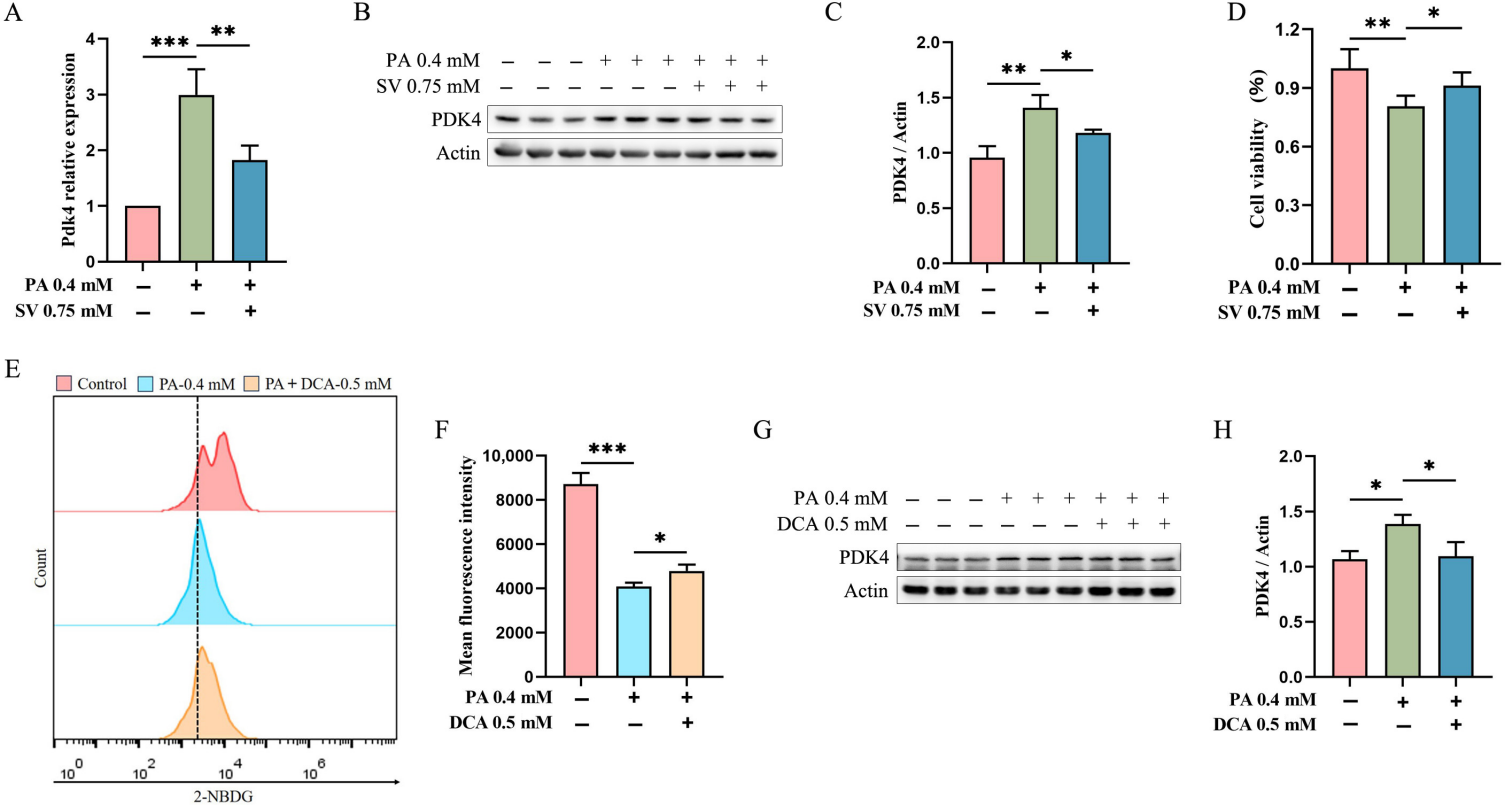
SV reduced PDK4 expression in PA‐induced C2C12 myotubes. (A) Quantitative analysis of *Pdk4* gene expression levels by qRT‐PCR. (B) PDK4 protein levels of C2C12 myotubes (differentiated for 3 d) that were co‐treated with 0.4 mM PA and 0.75 mM SV for 24 h were measured by western blotting. (C) Quantitative analysis of PDK4 protein from C2C12 myotubes that were co‐treated with 0.4 mM PA and 0.75 mM SV for 24 h. (D) Cell viability of C2C12 myotubes (differentiated for 3 d) that were co‐treated with 0.4 mM PA and 0.75 mM SV for 24 h was assessed with CCK‐8. (E) Glucose uptake capacity of C2C12 myotubes (differentiated for 3 d) that were co‐treated with 0.5 mM DCA and 0.4 mM PA for 24 h was measured with 2‐NBDG (DCA is a specific inhibitor of pyruvate dehydrogenase kinase [PDK]). (F) Quantitative analysis of the mean fluorescence intensity of glucose uptake of C2C12 myotubes that were co‐treated with 0.5 mM DCA and 0.4 mM PA for 24 h. (G) The PDK4 protein levels of C2C12 myotubes (differentiated for 3 d) that were co‐treated with 0.4 mM PA and 0.5 mM DCA for 24 h were detected by western blotting. (H) Quantification of PDK4 protein levels in PDK4 protein levels of C2C12 myotubes that were co‐treated with 0.4 mM PA and 0.5 mM DCA for 24 h. Each experiment was performed with three replications. Data are expressed as mean ± SD (*n* = 3). *p < 0.05, **p < 0.01, ***p < 0.001.

We observed that 0.75 mM SV significantly reduced *Pdk4* mRNA levels when compared with PA‐alone‐treated C2C12 myotubes (1.8 ± 0.6 vs. 3.0 ± 0.5 A.U., *p* = 0.019, Figure 2A). This finding was also confirmed by subsequent protein assays that showed a significantly reduced the level of PDK4 in 0.75 mM SV group (Figure 2B,C). Moreover, SV could significantly increase the cell viability of C2C12 myotubes when compared with the PA‐alone‐treated group (Figure 2D). We speculated that SV may improve cellular glucose uptake by reducing intracellular PDK4 levels. To test this hypothesis, we added 0.5 mM DCA (an inhibitor of PDK4) plus 0.4 mM PA to C2C12 myotubes for 24‐h treatment. When compared with the PA‐alone‐treated group, DCA was found to significantly promote cellular glucose uptake (Figure 2E,F) and reduce the level of PDK4 (Figure 2G,H).

### 3.3 SV Increased p‐AMPK and TBC1D1 Levels in PA‐Induced C2C12 Myotubes

**FIGURE 3:**
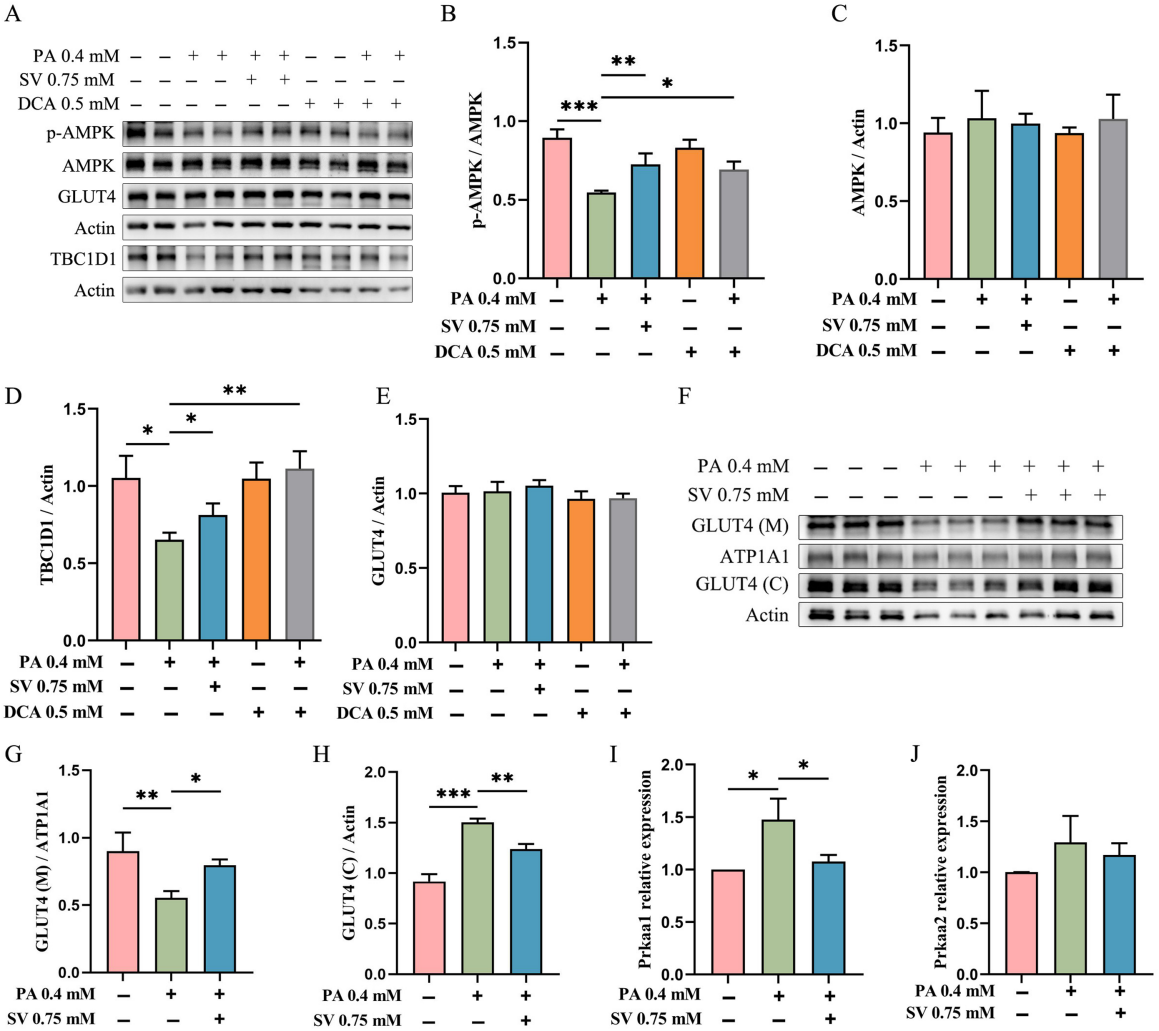
SV increased p‐AMPK and TBC1D1 levels in PA‐induced C2C12 myotubes. (A) The protein levels of C2C12 myotubes (differentiated for 3 d) that were treated with 0.4 mM PA, 0.5 mM DCA or 0.75 mM SV for 24 h were detected by western blotting. (B) Quantitative analysis of phospho‐AMPK levels of C2C12 myotubes that were treated with 0.4 mM PA, 0.5 mM DCA or 0.75 mM SV for 24 h. (C) Quantitative analysis of total AMPK levels of C2C12 myotubes that were treated with 0.4 mM PA, 0.5 mM DCA or 0.75 mM SV for 24 h. (D) Quantitative analysis of TBC1D1 levels of C2C12 myotubes that were treated with 0.4 mM PA, 0.5 mM DCA or 0.75 mM SV for 24 h. (E) Quantitative analysis of total GLUT4 levels of C2C12 myotubes that were treated with 0.4 mM PA, 0.5 mM DCA or 0.75 mM SV for 24 h. (F) The GLUT4 protein levels in the cell membrane and cytoplasm of C2C12 myotubes (differentiated for 3 d) that were co‐treated with 0.4 mM PA and 0.75 mM SV for 24 h were measured by western blotting. GLUT4 (M) is a GLUT4 protein located in the cell membrane. GLUT4 (C) is a GLUT4 protein located in the cytoplasm. (G) The GLUT4 protein levels in the cell membrane of C2C12 myotubes (differentiated for 3 d) that were co‐treated with 0.4 mM PA and 0.75 mM SV for 24 h were detected by western blotting. (H) The GLUT4 protein levels in the cytoplasm of C2C12 myotubes (differentiated for 3 d) that were co‐treated with 0.4 mM PA and 0.75 mM SV for 24 h were detected by western blotting. (I) Quantitative analysis of *Prkaa1* gene expression levels by qRT‐PCR. (J) Quantitative analysis of *Prkaa2* gene expression levels by qRT‐PCR. Each experiment was performed with three replications. Data are expressed as mean ± SD (*n* = 3). *p < 0.05, **p < 0.01, ***p < 0.001.

Since glucose is an intracellular energy donor and AMPK is a well‐known energy‐sensing factor, we subsequently explored whether SV could influence the AMPK expression. We observed that in the PA‐alone‐treated group, the levels of p‐AMPK and TBC1D1 were significantly reduced when compared with the control group, while total AMPK levels were not significantly altered (Figure 3A–D). By contrast, SV could significantly increase the levels of p‐AMPK and TBC1D1 when compared with the PA‐alone‐treated group (Figure 3A,B,D). Results from using DCA when compared with the PA‐alone‐treated group were largely similar to the findings of using SV (Figure 3A–D).

Compared with control group, neither PA, DCA nor SV could significantly alter total GLUT4 levels (Figure 3E). Moreover, in our post hoc experiments, we found that the GLUT4 level in the cell membrane of C2C12 myotubes in the SV group was significantly higher compared with the PA‐alone‐treated group, while the GLUT4 level in the cytoplasm in the SV group was significantly reduced (Figure 3F–H).

Surprisingly, increased mRNA levels of Prkaa1 but not Prkaa2 were observed in PA‐alone‐treated group, where Prkaa1 and Prkaa2 were two catalytic subunits of AMP‐activated protein kinase. SV had no effect on the mRNA levels of *Prkaa2*, but reduced the mRNA levels of *Prkaa1* when compared with the PA‐alone‐treated group (Figure 3I,J).

### 3.4 SV Decreased the Level of PDK4 and Increased the Levels of p‐AMPK and TBC1D1 in *Pdk4*‐overexpressing C2C12 Myotubes

**FIGURE 4:**
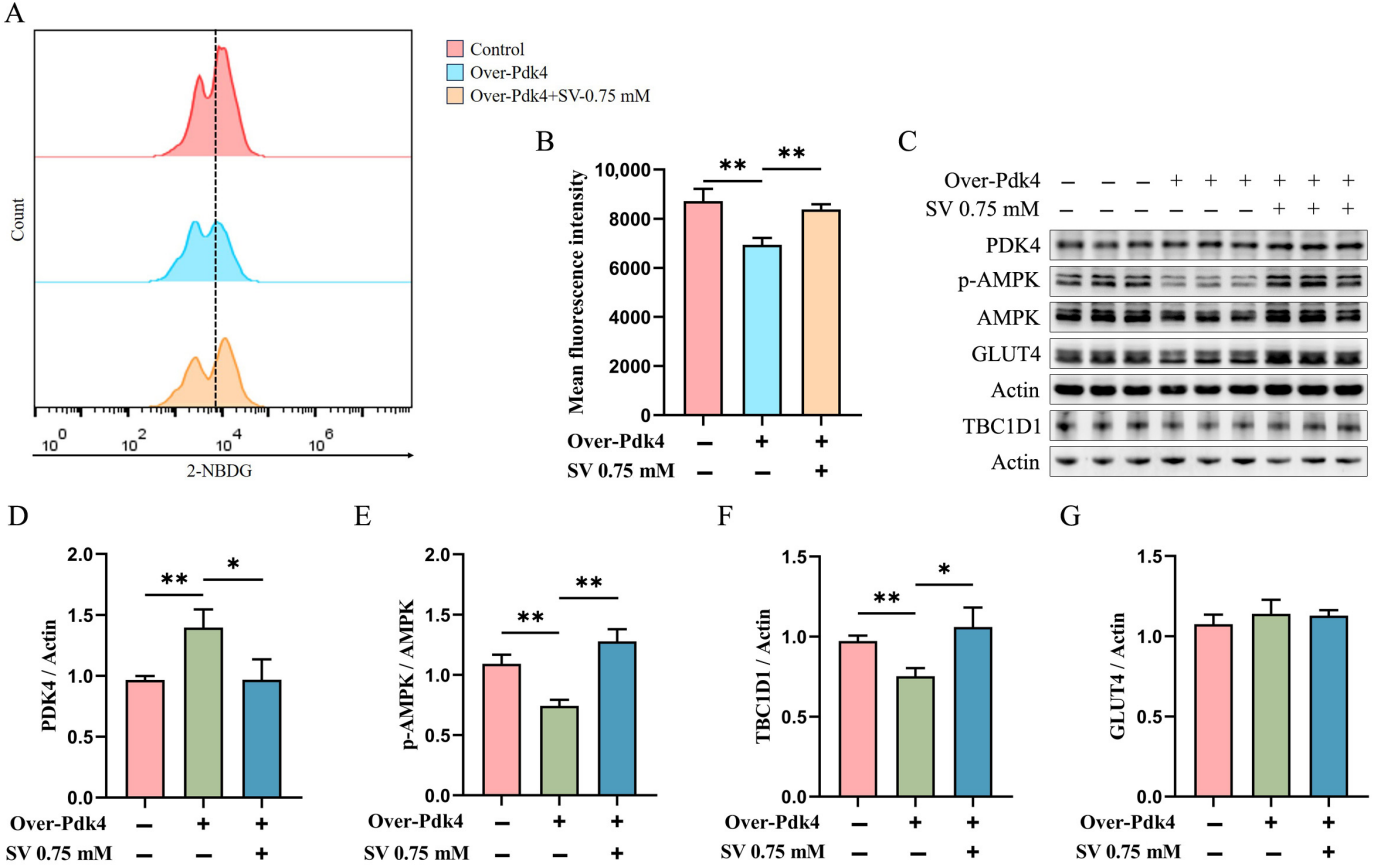
SV decreased the level of PDK4 and increased the levels of p‐AMPK and TBC1D1 in *Pdk4*‐overexpressing C2C12 myotubes. (A) Glucose uptake capacity of C2C12 myotubes (differentiated for 3 d) that were transfected with *Pdk4*‐overexpressing plasmid for 24 h was assayed by 2‐NBDG. (B) Quantitative analysis of the mean fluorescence intensity of glucose uptake capacity of C2C12 myotubes that were transfected with *Pdk4*‐overexpressing plasmid for 24 h. (C) The protein levels of C2C12 myotubes (differentiated for 3 d) that were transfected with *Pdk4*‐overexpressing plasmid for 24 h were detected by western blotting. (D) Quantitative analysis of the level of PDK4 of C2C12 myotubes (differentiated for 3 d) that were transfected with *Pdk4*‐overexpressing plasmid for 24 h. (E) Quantitative analysis of phospho‐AMPK levels of C2C12 myotubes (differentiated for 3 d) that were transfected with *Pdk4*‐overexpressing plasmid for 24 h. (F) Quantitative analysis of the level of TBC1D1 of C2C12 myotubes (differentiated for 3 d) that were transfected with *Pdk4‐*overexpressing plasmid for 24 h. (G) Quantitative analysis of the level of total GLUT4 of C2C12 myotubes (differentiated for 3 d) that were transfected with *Pdk4*‐overexpressing plasmid for 24 h. Each experiment was performed with three replications. Data are expressed as mean ± SD (*n* = 3). *p < 0.05, **p < 0.01, ***p < 0.001.

To further validate the effect of SV on PDK4 expression in C2C12 myotubes, *Pdk4*‐overexpressing plasmids were transfected into 3 d differentiated C2C12 myotubes and co‐treated with 0.75 mM SV for 24 h. Cellular glucose uptake was found to be significantly reduced after transfection with the *Pdk4*‐overexpressing plasmid when compared with the control group (empty vector transfection cells), whereas SV could significantly increase cellular glucose uptake (Figure 4A,B).

Pyruvate dehydrogenase kinase isozyme 4 levels in *Pdk4*‐overexpressing C2C12 myotubes were significantly increased when compared with the control group (Figure 4C,D). SV yielded a significantly reduced level of PDK4 and increased levels of p‐AMPK and TBC1D1 when compared with the *Pdk4*‐overexpressing group (Figure 4C–F). However, no significant effect of *Pdk4*‐overexpressing on total GLUT4 expression was found in C2C12 myotubes (Figure 4C,G).

## 4 Discussion

Currently, sugar‐free beverages have been increasingly consumed mainly to reduce calorie intake and prevent obesity and T2DM. While concerns about artificial sweeteners regarding their potential harmful effects on health have been increasingly raised [8, 10], SV as a natural sweetener is recognised as a safe sweetener with high sweetness and minimal calories. In this study, we found that SV significantly improved glucose uptake of PA‐treated skeletal muscle cells via the PDK4/AMPK/TBC1D1 pathway, further supporting its use in sugar‐free beverages.

It has been shown that acute oral administration of SV increased insulin sensitivity and facilitated insulin glucose transport in skeletal muscle of Zucker rats [28]. Although SV had no effect on the body weight of mice, it may have hypoglycaemic and renoprotective effects on the streptozotocin‐induced diabetes model [14]. For instance, SV could effectively inhibit oxidative stress in diabetic gastrocnemius muscle, promote glucose uptake and improve glucose tolerance in diabetic rats [29]. Nevertheless, evidence on the effects of SV on glucose uptake in skeletal muscles remained sparse and limited, making the mechanisms of SV for glucose uptake not fully understood.

Interestingly, we observed a significant effect of SV on glucose uptake by regulating PDK4 in a noninsulin‐related manner. Pyruvate dehydrogenase kinase isozyme 4 is an important enzyme in the tricarboxylic acid (TCA) cycle, in which PDK4 can inhibit PDH activity, reduce pyruvate oxidation and promote fatty acid synthesis [30]. Upregulation of PDK4 leads to increased fatty acid utilisation, thereby decreasing the utilisation of glucose [31]. Studies have shown the important regulatory role of PDK4 in the development of insulin resistance in skeletal muscles [19]. In line with previous studies, our research also identified that PDK4 played a key role in controlling glucose metabolism in PA‐induced C2C12 myotubes. Moreover, we found that SV significantly reduced the PDK4 levels and activated the AMPK/TBC1D1 signal to enhance glucose uptake. AMPK is an important factor in the regulation of intracellular energy homeostasis and glycolipid metabolism [32]. Intracellular PDK4 deficiency results in a decrease in ATP (adenosine triphosphate) levels, which could subsequently increase p‐AMPK levels [33]. This was consistent with our results that increased levels of PDK4 in C2C12 myotubes causing reduced levels of p‐AMPK. TBC1D1 is involved in the regulation of fatty acid oxidation and is also a key regulator of insulin‐ and exercise‐stimulated glucose uptake [34]. Exercise increased the level of TBC1D1 phosphorylation and increased AMPKα2 activity in skeletal muscles [35], where the AMPKα2 subunit is the major donor that regulates the AMPK activity of TBC1D1 Ser237 phosphorylation [36]. Moreover, TBC1D4 was found to regulate GLUT4 translocation from the cytoplasm to the plasma membrane as a substrate for AMPK [37].

In this study, we found that SV increased the levels of p‐AMPK and TBC1D1 in PA‐induced C2C12 myotubes, while the total GLUT4 levels were not altered. Although stevia extracts and derivatives were shown to increase the GLUT4 levels in skeletal muscles [38], no such changes in the total GLUT4 levels were observed in this study. However, in PA‐induced C2C12 myotubes, SV was found to promote GLUT4 translocation from the cytoplasm to the cell membrane, revealing that SV could enhance GLUT4 translocation rather than the quantity of GLUT4 level.

Interestingly, we also found that PA increased mRNA levels of *Prkaa1* in C2C12 myotubes. PRKAA1 (protein kinase AMP‐activated catalytic subunit α1) is a catalytic subunit of AMPK that plays a key role in the regulation of cellular energy metabolism [39]. *Prkaa1* also significantly gets involved in lipid metabolism in skeletal muscles, and *Prkaa1* deficiency results in delayed skeletal muscle development [40]. Therefore, we hypothesised that PA may have led to a compensatory increase in the mRNA level of *Prkaa1* in the C2C12 myotubes, whereas SV could reverse this increase of *Prkaa1* to improve glucose metabolism. However, PA reduced the level of p‐AMPK in C2C12 myotubes, but did not change the mRNA level of *Prkaa2*. Thus, the regulation of AMPK activity by PA may not depend on PRKAA1 or PRKAA2. Similarly, given the increased level of p‐AMPK and decreased mRNA level of *Prkaa1* in SV when compared with PA, the effects of SV on AMPK activity may also be independent of the two major subunits of AMPK (PRKAA1, PRKAA2), requiring more research for further exploration.

Although we found that SV could promote glucose uptake via the PDK4/AMPK/TBC1D1 pathway, we could not fully illustrate the detailed mechanism of regulating TBC1D1 by PDK4/AMPK. In addition, the specific molecular mechanisms involved in the regulation of GLUT4 transport from the cytoplasm to the cell membrane by TBC1D1 required further elucidation. Moreover, whether SV could interact with insulin‐related targets to modulate PDK4 and improve glucose uptake, warranted more research. Nevertheless, our study demonstrated that PDK4 may have the potential to serve as a molecular target for glucose uptake improvement, and SV may have beneficial effects on PDK4 and glucose uptake in skeletal muscle that was the most important organ for maintaining systemic glucose homeostasis. Even though these results required further investigation for validation, they may provide some evidence of SV as a safe natural sweetener for its use in sugar‐free beverages to prevent and control T2DM.

## 5 Conclusion

In conclusion, SV could ameliorate PA‐induced abnormal glucose uptake via the PDK4/AMPK/TBC1D1 pathway in C2C12 myotubes.

## Author Contributions

**Changfa Zhang:** Data curation (equal); Methodology (equal); Validation (equal); Writing – original draft (equal). **Shuai Li:** Formal analysis (equal); Methodology (equal); Writing – original draft (equal). **Likang Li:** Formal analysis (equal); Methodology (equal); Resources (equal); Writing – original draft (equal). **Ruoting Wang:** Formal analysis (equal); Investigation (equal); Methodology (equal); Writing – original draft (equal). **Shiming Luo:** Investigation (equal); Methodology (equal); Supervision (equal); Writing – original draft (equal). **Guowei Li:** Formal analysis (equal); Methodology (equal); Project administration (equal); Writing – review and editing (equal).

## Disclosure

None declared.

## Ethics Statement

Ethics approval was not required for this research.

## Conflicts of Interest

The authors declare no conflicts of interest.

## Data Availability

The data supporting the findings of this study are available from the corresponding author upon reasonable request.
